# Effects of partially replacing fishmeal with corn gluten meal on growth, feed utilization, digestive enzyme activity, and apparent nutrient digestibility for juvenile white shrimp, *Litopenaeus vannamei*

**DOI:** 10.3389/fvets.2023.1162599

**Published:** 2023-05-15

**Authors:** Huaxing Lin, Yingkai Deng, Dongwenjun Zhu, Qihui Yang, Xiaoqiu Zhou, Beiping Tan, Lin Feng, Shuyan Chi

**Affiliations:** ^1^College of Fisheries, Guangdong Ocean University, Zhanjiang, Guangdong, China; ^2^Aquatic Animals Precision Nutrition and High Efficiency Feed Engineering Research Center of Guangdong Province, Zhanjiang, Guangdong, China; ^3^Guangdong Provincial Key Laboratory of Aquatic Animal Disease Control and Healthy Culture, Zhanjiang, Guangdong, China; ^4^Institute of Animal Nutrition, Sichuan Agricultural University, Chengdu, China

**Keywords:** *Litopenaeus vannamei*, fishmeal, corn gluten meal, growth, apparent digestibility

## Abstract

This experiment was conducted to assess the possibility of replacing fishmeal (FM, Fishmeal content of the control group: 30%) with corn gluten meal (CGM) at the following levels: 0, 10, 20, 30, 40, and 60%. The experimental diets, formulated to be isonitrogenous and isocaloric, were studied for their effects on growth, feed utilization, digestive enzyme activity and apparent nutrient digestibility in juvenile white shrimps, *Litopenaeus vannamei* (initial mean weight = 0.71 ± 0.01 g). Seven hundred twenty healthy and uniformed-size shrimp were distributed to six groups of three replicates, each with 40 shrimp in each tank (0.5 m^3^). Each experimental diet was fed to shrimp four times daily to apparent satiation at 7:00, 11:30, 17:00, and 21:30, respectively, for 8 weeks. At the end of the experiment, the total weight of fish in each tank was weighed and randomly selected for testing, including fish nutrient composition and digestive enzyme activity. Results showed that no significant differences were observed in the weight gain rate (WGR), feed coefficient rate (FCR) and specific growth rate (SGR) of shrimp after 30% FM was replaced with CGM (*P* > 0.05), but these indicators significantly decreased at higher replacement rates. As CGM content increased, the content of crude protein and phosphorus in the shrimp decreased significantly (*P* < 0.05), whereas the crude fat content first increased significantly and then decreased (*P* < 0.05). Compared to the control group, the protease activity was significantly lower in the 40% group and the lipase activity was significantly lower in the 60% group (*P* < 0.05). Amylase activity was significantly increased with increasing CGM levels (*P* < 0.05). The digestibility of protein and lipid was significantly reduced by CGM replacement of more than 30% FM (*P* < 0.05). As CGM content increased, the digestion of energy and dry matter was first significantly increased and then significantly decreased (*P* < 0.05). In the 30, 40, and 60% groups, the digestibility of all amino acids (AA), except methionine (Met), arginine (Arg) and serine (Ser), was significantly lower than in the control group (*P* < 0.05). In summary, FM could be partially replaced by CGM in the feed of *L. vannamei*. Based on the broken-line regression analysis of WGR, the optimal dietary CGM replacement was 27.47%.

## 1. Introduction

The pacific white shrimp (*Litopenaeus vannamei*), commonly known as the South American white shrimp, has the advantages of large size, fast growth, and low nutritional requirements (1, 2). According to statistics, the annual production of *L*. *vannamei* in China exceeded one million tons, making it the country's most important crustacean-farmed animal (3). With the rapid development of the shrimp farming industry, the demand for shrimp feed has also increased yearly (4). However, the amount of fishmeal used in shrimp feed reached 30–40%, and the increase in feed costs had affected the development of shrimp farming in *L*. *vannamei* (5).

Fishmeal (FM) has been one of the most vital and ideal protein sources in feeds, with its good palatability, high protein content and balanced amino acid composition (6, 7). However, in recent years, due to overfishing, climatic phenomena, and other factors, world FM production has declined. FM prices have increased since they are in short supply while demand is increasing. There have been serious restrictions concerning the sustainable development of the aquatic feed industry (8, 9). Therefore, developing renewable alternative protein sources has been a key factor in the profitability of shrimp farming (10). Research into nutritious, cheap, readily available, highly efficient, and environmentally friendly plant protein sources to replace FM has become a hot topic for the aquafeed industry and academic research (11, 12).

Corn gluten meal (CGM) is one of the most widely used alternatives to FM and one of the most widely used plant protein sources (13). CGM is a by-product of maize processing, with high protein, low fiber, low anti-nutritional factors (ANF), and rich in minerals, vitamin B and vitamin E (14). It could be better absorbed by aquatic animals compared to other plant proteins and is an excellent plant-based protein. Moreover, it had great potential for exploitation due to its large scale of industrial production and low price (15). Studies have shown that CGM is an important alternative source of plant protein to FM as it has been widely used in replacing 15 to 75% of FM protein without affecting the growth performance and feed utilization capacity of *Takifugu fasciatus* (16), *Rachycentron canadum* (17), *Sparus aurata L*. (18), and *Dicentrarchus labrax* (19).

In addition, a wide range of aquatic researchers has conducted studies on the replacement of FM with CGM in the feed of *Macrobrachium rosenbergii* (20), *Macrobrachium nipponensis* (21) and *L*. *vannamei* (22–24), and the results showed that it was also feasible to replace FM with CGM in shrimp feed. However, most of the research on *L*. *vannamei* had evaluated the effect of CGM as an FM replacement in terms of growth and feed utilization, and relatively little had been reported on its digestibility. Digestive enzymes play a critical regulatory role in animal digestion, and researchers often judged the ability of aquatic animals to apply plant proteins based on their effect on digestive enzyme activity (25). Therefore, the present study aimed at evaluating the effects of replacing FM with CGM on the growth of juvenile *L. vannamei* and apparent nutrient digestibility.

## 2. Materials and methods

### 2.1. Diet formulation and preparation

The experiment included six isonitrogenous and isoenergetic diets with CGM replacing 0, 10, 20, 30, 40, and 60% FM protein, respectively (Table 1). All diet ingredients were ground through 60 mesh sizes and were thoroughly mixed homogenously in a V-type vertical mixer (JS-14S; Zhejiang Zhengtai Electric Co., Ltd.) (9, 26). Micro components, such as mineral premix and vitamin premix, were mixed by the progressive enlargement method. Lipids and water were then added to the premixed dry ingredients and thoroughly mixed to form a homogenous mixture (27). Then all mixture was pelleted (2.5 mm diameter) by making use of a double screw extruder (F-75; South China University of Technology, Guangzhou Guangdong, China) (28). After the prepared experimental feed was naturally dried to about 10% moisture, it was sealed in a vacuum-packed bag and stored at−20°C until it was fed (29, 30).

**Table 1:** Ingredients and proximate composition of the experimental diets (dry matter basis).

**Ingredients compositions (g·kg^−1^)**	**Diets**					
	**0%**	**10%**	**20%**	**30%**	**40%**	**60%**
Fish meal (FM)	300.00	270.00	240.00	210.00	180.00	120.00
Corn gluten meal (CGM)	0.00	31.40	62.90	94.40	125.90	188.80
Fish oil	13.50	15.50	17.00	18.50	20.00	24.50
L-Lys	0.10	1.10	2.30	3.50	4.80	7.40
DL-Met	1.40	1.40	1.50	1.50	1.50	1.50
L- Arg	0.00	0.10	0.10	0.30	0.70	1.00
L-Thr	0.00	0.30	0.70	1.20	1.60	2.60
Cellulose	30.80	26.00	21.30	16.40	11.30	0.00
Others^*^	637.20	637.20	637.20	637.20	637.20	637.20
Mineral mixture^**^	10.00	10.00	10.00	10.00	10.00	10.00
Vitamin mixture^***^	2.00	2.00	2.00	2.00	2.00	2.00
Chromic oxide (Cr_2_O_3_)	5.00	5.00	5.00	5.00	5.00	5.00
Totals	1000.00	1000.00	1000.00	1000.00	1000.00	1000.00
**Nutrition composition**						
Moisture (g·kg^−1^)	97.20	94.00	97.00	90.20	93.60	91.70
Crude protein (g·kg^−1^)	401.50	403.60	404.90	402.20	406.40	405.90
Crude lipid (g·kg^−1^)	87.60	86.50	86.60	85.20	85.40	86.50
Gross energy (MJ·kg^−1^)	19.28	19.41	19.69	19.64	19.51	19.56

* Others: Soybean meal 200 g, Peanut meal 120 g, Wheat gluten meal 30 g, Shrimp shell meal 30 g, Wheat flour 226.6 g, Lecithin 15 g, Mono calcium phosphate 15 g, Ascorbic acid poly phosphate 0.3 g, Choline chloride 0.3 g.

** Mineral premix consisted of (g·kg^−1^ premix): Ferric citrate 13.71 g, ZnSO_4_·7H_2_O 28.28 g, MgSO_4_·7H_2_O 0.12 g, MnSO_4_·H_2_O 12.43 g, CuSO_4_·5H_2_O 19.84 g, CoC_*l*2_·6H_2_O 4.07 g, KIO_4_ 0.03 g, KCl 15.325 g, Na_2_SeO_3_ 0.02 g, and Cellulose 906.18 g.

*** Vitamin premix consisted (g·kg^−1^ premix): Thiamine hydrochloride 25.50 g, Riboflavin 25.0 g, Pyridoxine hydrochloride 50.0 g, Cyanocobalamin 0.1 g, Menadione 5.0 g, All-trans-a-tocopherol acetate 99.0 g, Retinyl acetate 10.0 g, Vitamin D 50 g, Nicotinic acid 101.0 g, D-Ca-pantothenate 61.0 g, Biotin 25.0 g, Folic acid 6.25 g, Inositol 153.06 g, Cellulose 383.44 g.

### 2.2. Experimental animals and breeding management

The experiment was conducted in an indoor flow through an aquarium system of Guangdong Yuehai Feed Group Company Limited, Zhanjiang, China. Juvenile shrimp were obtained from the Guangdong Yuehai Feed Group Company Limited shrimp farm and kept in eighteen 0.5 m^3^ circular fiberglass tanks for 10-day acclimatization. At the beginning of the experiment, the acclimated experimental shrimp (initial mean weight = 0.71 ± 0.01 g) were randomly distributed into cylindrical fiber-glass tanks at the stocking rate of 40 shrimp per tank. Tanks were supplied with flowing filtered seawater with a flow rate of ~1.0 L·min^−1^ with adequate aeration. Each experimental diet was fed to shrimp four times daily to apparent satiation at 7:00, 11:30, 17:00, and 21:30, respectively (31). Seawater temperature and salinity were monitored twice daily between 9:00 and 15:00. Uneaten feed particles and feces were removed by siphoning (32). During the experimental period, the temperature was 28.0–30.5°C, salinity was 29.0–30.0, dissolved oxygen was 6.5–7.0 mg·L^−1^, and total ammonia nitrogen was 0.3–0.5 mg·L^−1^. The feeding trial lasted 8 weeks.

### 2.3. Sample collection

Shrimp were starved for 24 h prior to collection at the cessation of the trial and anesthetized afterward with MS-222 (1:10,000) (8). The shrimp samples of each experimental group were calculated and weighed, and it was determined the weight gain rate, survival rate, specific growth rate, and feed coefficient rate (33). Afterward, three shrimp samples per tank were randomly selected to store at −20°C to detect the whole-body composition (34).

The ingredients of the experimental diets and shrimp samples (crude protein, crude lipid, moisture and ash) were measured by using standard methods AOAC (35, 36). The content of crude protein was assayed by means of Kjeldahl method (N × 6.25) (37) and crude lipid was measured using the method of Soxhlet extraction (38). The content of ash was measured by a muffle furnace instrument burning at 550°C for 12 h (39).

#### 2.3.1. Preparation of crude enzyme solution

At the end of the feeding trial, the hepatopancreas of 15 shrimps was randomly taken from each diet group after 24 h of starvation (40). The samples were weighed, added 10 times the volume of pre-cooled deionized water at 4°C, homogenized, and centrifuged at 4 000 r·min^−1^ for 10 min (41, 42). The supernatant was taken as the crude enzyme solution and stored at 4°C for testing.

#### 2.3.2. Fecal collection

Half an hour after feeding, the rearing tanks and collection column were brushed out to remove uneaten feed and fecal residues (43). Fecal samples were collected four times daily for each tank (07:30, 12:00, 17:30, and 22:00). Fecal collected from the settling columns were immediately collected on filter paper for 60 min at 4°C and stored at−20°C for chemical analyses. Daily fecal samples from each tank were pooled together throughout the experiment until a sufficient sample (approximately 10 g per pool) was obtained for chemical analysis (44).

### 2.4. Analysis of samples and data statistics

#### 2.4.1. Determination of digestive enzyme activity

The digestive enzyme activities and tissue protein content were determined by using a detection kit (Nanjing Jian Cheng Bioengineering Institute, China). Proteinase activity was measured by the xanthine oxidase method, according to Lin et al. (45). The amylase activity was measured by Liu et al. (46). Lipase activity was determined in hepatopancreas using a full band enzyme marker (Uquant, BioTek, USA) (47).

#### 2.4.2. Calculation of digestibility

Gross energy was determined by using an adiabatic bomb calorimeter (C2000, IKA, Germany) (48). Chromic oxide and phosphorus content of diets and feces were determined by ICP atomic emission Spectrophotometry [IRIS Advantage (HR), Thermo Jarrell Ash, Woburm, USA]. Amino acid concentrations in the experimental diets and fecal material were determined with an automatic amino acid analyzer (Hitachi Model 835-50, Japan) equipped with a column for physiological fluid analysis (2.6–150 mm, Hitachi custom ion-exchange resin No. 2619).

Apparent digestibility coefficients (ADCs) of nutrients and energy for the reference and test diets were calculated by the indicator method (49):

ADC of dry matter (%) = 100 × [1 - (dietary Cr_2_O_3_)/ fecal Cr_2_O_3_]

ADC of nutrients or energy (%) = 100 × [1 - (F/D × D_Cr_ / F_Cr_)]

Where F was the percent of nutrients or energy in feces, D was the percent of nutrients or energy in the diet, D_Cr_ was the percent of chromic oxide in the diet, and F_Cr_ was the percent of chromic oxide in feces.

The ADCs for dry matter, crude protein, crude lipid, phosphorus, energy, and amino acids (AA) were calculated from the respective digestibility coefficients for the reference diet and test diets based on the 30% substitution of test ingredients in the reference diet (50).

#### 2.4.3. Growth parameters were calculated as follows

Weight gain rate (WGR, %) = 100 × (final mean weight-initial mean weight)/initial mean weight;

Specific growth rate (SGR, %·d^−1^) = 100 × [ln (final weight / initial weight)] / days of the experiment;

Feed coefficient rate (FCR) = feed consumed (dry weight) / body weight gain;

Survival rate (SR, %) = 100 × (final shrimp number) / (initial shrimp number).

#### 2.4.4. Statistical analysis

Results were expressed as mean ± SD. Data were analyzed as a design using the SPSS^®^ version 13.0 (Chicago, IL, USA). One-way ANOVA was performed. Duncan's multiple range test was used to identify significant differences among digestibility coefficients, and *P* < 0.05 was considered a statistically significant difference.

## 3. Results

### 3.1. Effects of CGM on growth performance for *L. vannamei*

The growth performance and feed utilization for juvenile *L. vannamei* are shown in Table 2. WGR and SGR significantly decreased as the level of replacement of FM with CGM increased at levels up to 40% (*P* < 0.05). FCR significantly increased when FM replacement level with CGM was over 40% (*P* < 0.05). Shrimp WGR ranged from 1201.55 to 1542.64%, and FCR from 1.19 to 1.47 for all dietary groups at the end of 8 weeks were recorded.

**Table 2:** Effect of partial replacement of FM with CGM on growth and feed utilization for *L*. *vannamei*.

**Diets**	**Initial weight (g)**	**Final weight (g)**	**WGR (%)**	**FCR**	**SGR (%·d^−1^)**	**SR (%)**
0%	0.71 ± 0.003	11.73 ± 0.33^a^	1542.64 ± 55.55^a^	1.20 ± 0.04^c^	4.99 ± 0.06^a^	96.66 ± 3.81
10%	0.71 ± 0.002	11.35 ± 0.09^a^	1485.70 ± 15.36^a^	1.25 ± 0.03^bc^	4.93 ± 0.01^a^	97.50 ± 2.50
20%	0.71 ± 0.001	11.39 ± 0.47^a^	1497.86 ± 67.13^a^	1.19 ± 0.04^c^	4.94 ± 0.07^a^	99.16 ± 1.44
30%	0.71 ± 0.004	11.22 ± 0.26^a^	1461.84 ± 27.21^a^	1.20 ± 0.02^c^	4.90 ± 0.03^a^	100.00 ± 0.00
40%	0.71 ± 0.003	10.28 ± 0.49^b^	1340.33 ± 65.72^b^	1.35 ± 0.10^b^	4.76 ± 0.08^b^	96.66 ± 2.50
60%	0.71 ± 0.003	9.28 ± 0.21^c^	1201.55 ± 25.72^c^	1.47 ± 0.03^a^	4.58 ± 0.03^c^	100.00 ± 0.00

Data represented mean ± SD (*n* = 3). In the same column, values with different superscripts mean significant difference (*P* < 0.05), values with the same superscripts mean insignificant difference (*P* > 0.05). WGR, Weight gain rate; FCR, Feed coefficient rate; SGR, Specific growth rate; SR, Survival rate.

**Figure 1:**
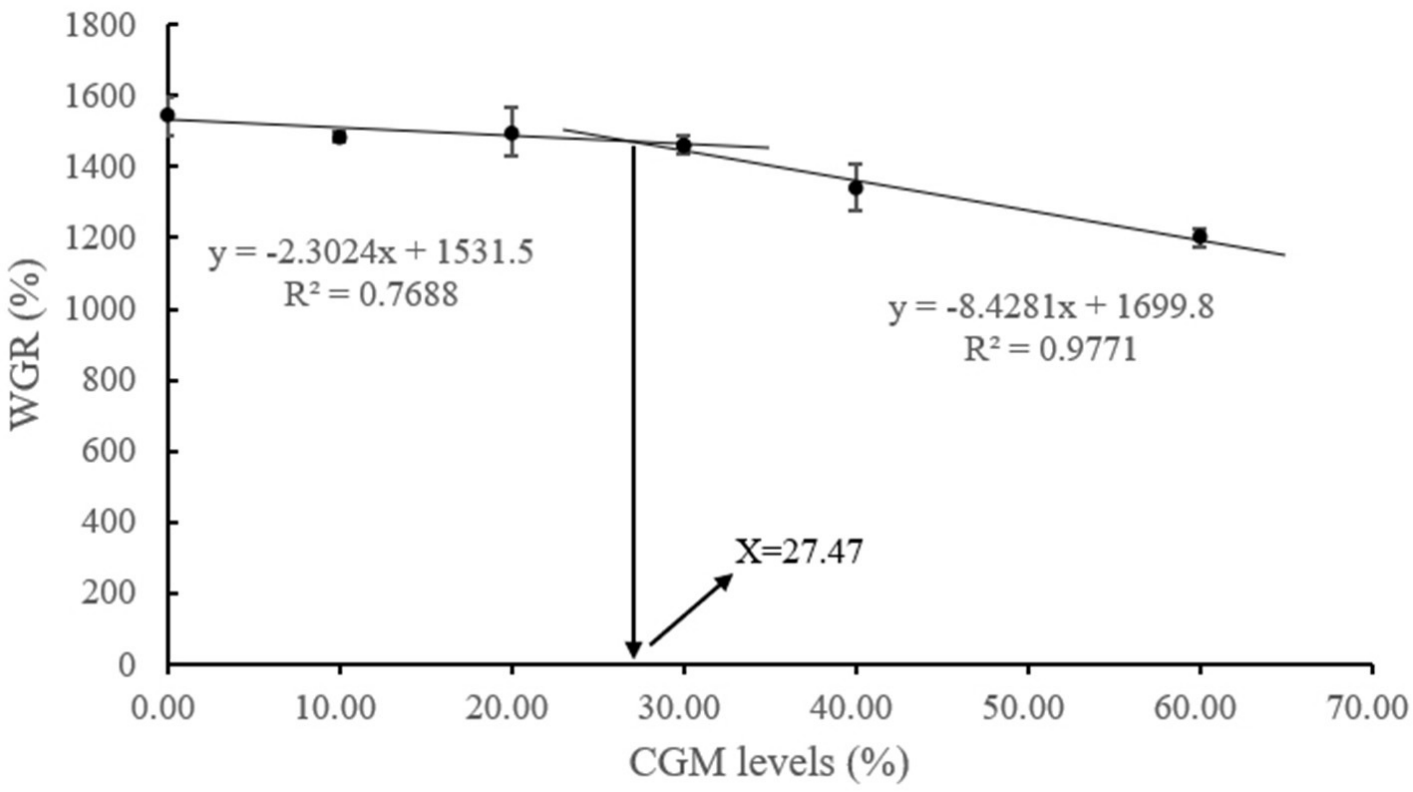
Based on broken-line regression analysis between WGR and dietary CGM replacement levels (y = −2.3024x + 1531.5, *R*^2^ = 0.7688; y = −8.4281x + 1699.8, *R*^2^ = 0.9771), the optimum replacement level of dietary CGM was estimated to be 27.47%.

With WGR as the basis for judgement and by fitting it with a broken line model (Figure 1), the optimum replacement level of FM to be replaced by CGM was 27% (Y = −8.4281x + 1699.8, *R*^2^ = 0.98; Y = −2.3024x + 1531.5, *R*^2^ = 0.77).

### 3.2. Effects of CGM on body compositions for *L. vannamei*

Whole-body proximate composition of shrimp is presented in Table 3. The crude protein content of shrimp was highest in the 0% group, followed by the 20% group, and significantly higher in both groups than in the 30, 40, and 60% groups (*P* < 0.05). The crude fat content was highest in the 60% group and lowest in the 0% group (*P* < 0.05). The crude fat was significantly lower in the 0% group than in the 60% group (*P* < 0.05) and not significantly different from the 10 and 40% groups (*P* > 0.05). The phosphorus content was highest in the control group, which was significantly higher than that in the 40 and 60% groups (*P* < 0.05). No significant difference was found in the ash content (*P* > 0.05).

**Table 3:** Effect of partial replacement of FM with CGM on whole body composition for *L*. *vannamei*.

**Diets**	**Crude protein (%)**	**Crude lipid (%)**	**Crude ash (%)**	**Phosphorus (%)**
0%	70.84 ± 0.45^a^	11.31 ± 0.28^c^	11.27 ± 0.66	1.00 ± 0.06^a^
10%	69.28 ± 0.53^ab^	12.52 ± 0.18^bc^	10.57 ± 1.47	0.85 ± 0.04^ab^
20%	69.75 ± 0.37^a^	14.19 ± 0.69^ab^	10.85 ± 0.33	0.93 ± 0.08^ab^
30%	66.72 ± 1.24^c^	11.67 ± 0.16^c^	11.45 ± 0.61	0.91 ± 0.03^ab^
40%	67.03 ± 2.83^bc^	11.60 ± 2.35^c^	11.61 ± 0.62	0.86 ± 0.03^b^
60%	66.90 ± 1.17^bc^	15.64 ± 0.52^a^	11.29 ± 0.16	0.88 ± 0.09^b^

Data represented mean ± SD (*n* = 3). In the same column, values with different superscripts mean significant difference (*P* < 0.05), values with the same superscripts mean insignificant difference (*P* > 0.05).

### 3.3. Effects of CGM on digestive enzyme activities for *L. vannamei*

No significant difference in protease activity in hepatopancreas was found at substitution levels of 0–30 and 60%. When substitution levels were 40%, protease activity in hepatopancreas decreased significantly with increasing substitution levels (*P* < 0.05, Table 4). In hepatopancreas, the pattern of change in lipase activity was in line with that of protease activity. At substitution levels of 0–30%, lipase activity in hepatopancreas increased and then decreased with increasing substitution levels, with the highest being in the 10% group. At substitution levels of 40–60%, protease activity in hepatopancreas gradually decreased. Lipase activity in hepatopancreas was significantly higher in the 10% group than in the 30, 40, and 60% groups (*P* < 0.05). In hepatopancreas, amylase activity increased significantly with increasing substitution levels (*P* < 0.05). Amylase activity in the 30, 40, and 60% groups were significantly higher than in the 0 and 10% groups (*P* < 0.05).

**Table 4:** Effect of partial replacement of FM with CGM on digestive enzyme activities in hepatopancreas for *L*. *vannamei*.

**Diets**	**Protease activity (U·mg^−1^)**	**Lipase activity (U·mg^−1^)**	**Amylase activity (U·mg^−1^)**
0%	172.36 ± 2.22^ab^	11.71 ± 2.63^ab^	1.02 ± 0.01^c^
10%	168.69 ± 5.09^ab^	14.76 ± 3.87^a^	1.10 ± 0.01^c^
20%	167.11 ± 12.17^ab^	11.15 ± 2.61^abc^	1.33 ± 0.01^ab^
30%	172.80 ± 7.99^a^	9.65 ± 1.37^bc^	1.34 ± 0.02^a^
40%	150.83 ± 3.58^c^	7.78 ± 1.51^bc^	1.31 ± 0.03^b^
60%	164.70 ± 5.40^bc^	7.37 ± 1.80^c^	1.34 ± 0.03^a^

Data represented mean ± SD (*n* = 3). In the same column, values with different superscripts mean significant difference (*P* < 0.05), values with the same superscripts mean insignificant difference (*P* > 0.05).

### 3.4. Effects of CGM on apparent digestibility coefficients for *L. vannamei*

Apparent digestibility coefficients (ADCs) of dry matter, protein, lipid, energy, phosphorus, and AA in the experimental diets for juvenile *L. vannamei* were shown in Tables 5, 6, respectively. The ADCs of dry matter, protein, lipid, energy, and AA were significantly affected (*P* < 0.05) by replacing levels of FM with CGM. No significant difference was found in the ADCs of phosphorus (ranging from 92.94 to 93.88%). ADCs of crude protein in the 30, 40, and 60% groups significantly decreased as the replacing levels of FM with CGM increased (*P* < 0.05), and it was significantly lower than that recorded in the remaining groups (0–20%). ADCs of energy in the 0% group (83.54 ± 0.34%) were significantly lower than those in the 10 and 20% groups (85.29 ± 0.08 and 86.60 ± 0.36%, respectively) (*P* < 0.05). ADCs of lipids in the 30, 40, and 60% groups (97.01 ± 4.30 %) were significantly lower than those in the 0 and 10% groups (*P* < 0.05), and no significant difference in ADCs of lipids was found between the 0 and 10% groups (*P* > 0.05).

**Table 5:** Effect of partial replacement of FM with CGM on apparent digestibility of nutrients and energy for *L*. *vannamei*.

**Diets**	**Protein (%)**	**Lipid (%)**	**Phosphorus (%)**	**Energy (%)**	**Dry matter (%)**
0%	91.73 ± 0.17^a^	97.01 ± 4.30^a^	92.35 ± 0.19	83.54 ± 0.34^b^	72.43 ± 0.56^c^
10%	91.69 ± 0.14^a^	95.17 ± 0.94^a^	92.94 ± 0.62	85.29 ± 0.08^a^	76.09 ± 0.42^b^
20%	91.79 ± 0.31^a^	92.66 ± 0.99^abc^	93.88 ± 0.39	86.60 ± 0.36^a^	79.09 ± 0.88^a^
30%	85.72 ± 0.46^b^	90.39 ± 5.48^bc^	93.31 ± 0.49	82.05 ± 0.38^c^	75.12 ± 2.11^b^
40%	85.29 ± 1.21^b^	89.62 ± 3.83^bc^	92.67 ± 0.10	81.84 ± 1.51^c^	72.83 ± 0.74^c^
60%	84.45 ± 1.32^b^	88.34 ± 0.39^c^	93.26 ± 0.67	80.25 ± 0.99^d^	71.80 ± 1.11^c^

Data represented mean ± SD (*n* = 3). In the same column, values with different superscripts mean significant difference (*P* < 0.05), values with the same superscripts mean insignificant difference (*P* > 0.05).

**Table 6:** Effect of partial replacement of FM with CGM on apparent digestibility of amino acid for *L*. *vannamei*.

**Amino acids composition**	**0%**	**10%**	**20%**	**30%**	**40%**	**60%**
**Essential amino acids**						
Lysine	95.39 ± 0.25^a^	95.66 ± 0.66^a^	95.93 ± 0.47^a^	94.49 ± 0.55^b^	94.31 ± 0.50^b^	94.38 ± 0.40^b^
Methionine	78.24 ± 3.98^d^	82.98 ± 0.66^b^	87.95 ± 1.46^a^	85.97 ± 0.43^ab^	84.36 ± 0.83^bc^	82.86 ± 1.31^c^
Isoleucine	93.92 ± 0.08^a^	92.93 ± 0.44^a^	92.70 ± 0.79^a^	88.71 ± 0.99^b^	87.31 ± 1.00^b^	85.30 ± 1.44^c^
Leucine	94.55 ± 0.13^a^	94.91 ± 0.42^a^	92.70 ± 1.17^a^	87.06 ± 1.62^b^	85.11 ± 1.66^b^	81.43 ± 1.64^c^
Phenylalanine	93.56 ± 0.14^a^	94.39 ± 0.25^a^	94.13 ± 0.82^a^	90.81 ± 0.50^b^	90.19 ± 0.62^b^	88.50 ± 0.81^c^
Valine	90.63 ± 0.53^a^	89.88 ± 0.30^a^	89.91 ± 0.30^a^	86.12 ± 0.73^b^	85.34 ± 0.14^bc^	84.32 ± 1.49^c^
Histidine	95.22 ± 0.05^a^	95.22 ± 0.18^a^	96.19 ± 0.52^a^	93.52 ± 0.67^b^	93.72 ± 0.85^b^	93.17 ± 0.58^b^
Arginine	96.91 ± 0.39^bc^	97.42 ± 0.18^ab^	97.71 ± 0.08^a^	96.63 ± 0.49^c^	96.67 ± 0.36^c^	97.27 ± 0.32^ab^
Threonine	91.28 ± 0.55^a^	91.34 ± 0.42^a^	92.73 ± 1.47^a^	88.45 ± 1.15^b^	87.77 ± 0.93^b^	87.96 ± 1.04^b^
**Non-essential amino acids**						
Aspartic	93.22 ± 0.38^a^	92.47 ± 0.26^a^	93.31 ± 0.76^a^	89.98 ± 0.33^b^	89.48 ± 0.35^b^	89.30 ± 1.36^b^
Tyrosine	94.29 ± 0.08^a^	94.81 ± 0.28^a^	95.46 ± 0.61^a^	91.85 ± 0.88^b^	91.93 ± 1.23^b^	91.03 ± 0.90^b^
Serine	92.13 ± 0.50^ab^	92.85 ± 0.29^a^	93.93 ± 0.84^a^	90.36 ± 1.57^b^	90.08 ± 2.19^b^	90.61 ± 1.30^b^
Glutamic	95.56 ± 0.06^a^	95.16 ± 0.15^a^	94.77 ± 1.69^a^	89.91 ± 0.73^b^	89.75 ± 1.00^b^	87.78 ± 1.26^c^
Proline	93.81 ± 0.51^a^	94.54 ± 1.30^a^	94.51 ± 2.04^a^	89.25 ± 1.92^b^	89.58 ± 2.29^b^	87.95 ± 0.86^b^
Glycine	89.42 ± 0.23^b^	88.87 ± 0.15^b^	90.85 ± 1.05^a^	86.48 ± 0.60^c^	85.71 ± 0.42^c^	85.88 ± 1.04^c^
Alanine	91.60 ± 0.48^a^	91.21 ± 0.49^a^	88.51 ± 0.73^b^	82.79 ± 1.42^c^	81.25 ± 1.76^c^	78.71 ± 2.23^d^
Total amino acids	93.49 ± 0.17^a^	93.57 ± 0.15^a^	93.59 ± 0.91^a^	89.68 ± 0.47^b^	89.02 ± 0.65^b^	87.70 ± 1.12^c^

Data represented mean ± SD (*n* = 3). In the same column, values with different superscripts mean significant difference (*P* < 0.05), values with the same superscripts mean insignificant difference (*P* > 0.05).

Apparent amino acid availability (AAAA) declined with the incorporation of CGM, reflecting the ADC of protein. In digestibility of essential AA, most AAAA values in the 0, 20, and 30% groups (*P* < 0.05) were significantly higher than that in the other groups (*P* < 0.05), and the lowest values were observed in the 40 and 60% groups, except Arg and Met. Met digestibility was highest in the 20% group and lowest in the 0% group, significantly lower than the rest of the groups (*P* < 0.05). The difference in Met digestibility between the 20 and 30% groups was not significant (*P* > 0.05) but significantly higher than the rest of the groups (*P* < 0.05). Among the non-essential amino acids (NEAA), the apparent digestibility of aspartic, tyrosine, glutamic, and proline were not significantly different in the 10, 20, and 30% groups (*P* > 0.05), all of which were significantly higher than the rest of the groups (*P* < 0.05). No significant difference in ADCs of glycine and alanine were found between the 0 and 10% groups (*P* > 0.05), and no significant differences in ADCs of serine was found between all groups (*P* > 0.05). The apparent digestibility of total amino acids (TAA) followed the same trend as that of NEAA, but the apparent digestibility of TAA was significantly higher in the 30 and 40% groups than in the 60% group (*P* < 0.05), whereas the differences between the other groups, were not significant (*P* > 0.05).

## 4. Discussion

CGM is a plant-based ingredient with high protein content, low ANF and low fiber (15). As a low-cost protein source, CGM has been investigated in a variety of aquaculture species (14, 51, 52). Some encouraging results had been obtained in shrimps. In *M. rosenbergii* (initial mean weight = 1.35 ± 0.06 g), the replacement of 18.20% FM (the control group fishmeal content: 33%) with CGM showed that no significant differences were found in the WGR and FCR (20). In addition, in terms of WGR, SR, and FCR, it was feasible to replace 44.4% of FM with CGM in feed formulations (the control group fishmeal content: 27%), and no significant difference was observed in the overall growth performance of *M. nipponensis* (initial mean weight = 0.23 ± 0.12 g) in the mid to late stages (21).

In the present study, no significant differences were found in WGR and FCR after the replacement of 30% FM (fishmeal content of the control group: 30%) with CGM for *L*. *vannamei*, whereas the replacement of more than 30% FM showed a significant decrease in WGR. A possible explanation for this decrease may be that fishmeal contains unknown growth-promoting factors and CGM is less palatable than fishmeal (51). Therefore, the higher the amount of CGM replacement for FM, the greater the effect on the growth of *L*. *vannamei*, which might also be an essential reason for the significantly higher FCR. Based on the present results, the optimal CGM replacement level of *L*. *vannamei* was 27.47%, as predicted by the WGR model. However, Yao et al. (24) showed that the replacement of FM protein (the control group fishmeal content: 27%) with CGM could reach 15% without significantly affecting the growth performance of *L*. *vannamei* (initial mean weight = 0.147 ± 0.01 g). In addition, Han et al. (23) investigated the possibility of replacing 17.2% of FM (the control group fishmeal content: 35%) with CGM based on the growth performance and feed factor of *L*. *vannamei* (initial mean weight = 0.0136 ± 0.10 g). The conclusions of the above studies differed from the present study, probably because the optimum amount of replacement varies according to species or body-weight size (20), but all showed that CGM could be used as an alternative to FM in shrimp feed.

Body composition directly reflected animal growth and indirectly reflected feed quality (8). Studies on *M. rosenbergii* (20) and *M. nipponensis* (21) had shown that no significant difference in shrimp composition was observed with the increase in CGM content. In addition, Han et al. (23) also showed no significant difference in the whole-body composition after the partial replacement (15%) of FM with CGM for *L. vannamei*. However, in the present study, the protein content of the shrimp was significantly reduced when the proportion of CGM substituted for FM was too high (30%). This was due to the poor AA balance of the CGM itself (53) and the poor palatability of feeds containing high proportions of CGM (54), resulting in the low digestibility of AA by shrimp. Therefore, the crude protein content of whole shrimp was significantly lower, in line with the growth performance of shrimp. In addition, as the amount of FM replaced with CGM increased, shrimp body fat content first increased significantly and then decreased in the present study. The increase in body fat content might be related to a deficiency of the ketogenic amino acid, lysine (Lys), in the protein source leading to accelerated catabolism of other AA, generating large amounts of carbon chains and glucose substrates required for fat synthesis and increased activity of fatty acid synthase in the fish, thus promoting fat synthesis (55). In the present study, the experimental feed was not deficient in Lys and had been supplemented with crystal amino acids; however, the digestibility of Lys by the shrimps in the experimental group was low. Furthermore, the effective use of crystalline amino acids by shrimps has been debated, focusing on issues such as synchronization of amino acid absorption, differences in feed species and leaching in water (56). In addition, CGM is poorly palatable and excessive levels in feed may affect nutrient intake (54). Therefore, high proportions of CGM could affect the effective use of AA by *L*. *vannamei*.

Digestive enzyme activity is an important indicator of an animal's digestive function and determines its ability to digest nutrients, thus affecting the rate of growth and development (57). Digestive enzyme activity determined the ability of shrimps to digest and absorb nutrients, with higher enzyme activity indicating greater digestive capacity (58). Numerous studies have shown that plant proteins significantly reduce protease activity in aquatic animals such as *Cyprinus carpio* (59), *Gadus morhua* (60) and juvenile tilapia (*Oreochromis niloticus* × *O. aureus*) (61), among others. In the present study, no significant difference in the proteinase activity was found after the partial replacement of FM with CGM for *L*. *vannamei*. This conclusion was in line with the study on *M. rosenbergii* (56). However, protease activity was significantly reduced when the proportion of CGM replacing FM was above 40%. In *Oncorhynchus mykiss* and *Sparus aurata* (62), protease activity decreased with increasing amounts of plant protein sources replacing FM. This was because plant-derived feeds may contain ANF, such as trypsin inhibitors (63), saponins (64), and phytoalexins (65) that affect the secretion of proteases in aquatic animals.

Lipase was an inducible extracellular enzyme whose activity was related to the lipid content of the feed consumed (66). It was found that the lipase activity in the digestive tract of *Larimichthys crocea* remained low during development, presumably due to the consumption of bait, mainly fresh bait with very low-fat content and some compound feed (67). In addition, while being higher in the forage and domesticated groups of *Pelteobagrus vachelli* than in the live bait group, was observed as positively correlating with the fat in the bait (68). However, there was also experimental evidence that the lipase activity was reduced in high-fat content baits, such as juvenile *Pagrosomus major* (69). In the present study, the lipase activity increased first and later-decreased with increasing CGM inclusion. In addition, no difference in the fat content of the experimental diets was found. The present study is in agreement with the study of Zhong et al. (70) on *Takifugu obscurus*. The differences in lipase activity may be due to the different sources of fat in the diets of the groups. Therefore, the differences in lipase activity need further confirmation.

Amylase activity is related to food habits (71). Agrawal et al. (72) investigated the differences in amylase in carnivorous, omnivorous, and herbivorous aquatic animals and deduced that herbivorous aquatic animals had an intense amylase activity and carnivorous aquatic animals the weakest. Studies on *Ctenopharyngodon Idella* (73), *P. vachelli* (74), and *Ietalurus punetaus* (75) have shown that increased plant protein content in feed increased the activity of amylase in aquatic animals. In addition, it was found that amylase activity was positively correlated with the starch content in the feed (69). In *Scophthalmus maximus* (76), amylase activity increased with increasing soybean meal content. In the present study, amylase activity was improved in all experimental groups compared to the control group as the amount of CGM replacement increased, indicating that CGM increased amylase activity in shrimp.

Apparent digestibility allowed evaluation of the feeding potential of protein sources (17). Studies have shown that the apparent digestibility of crude protein in *Psetta maxima* (22) gradually decreased with CGM increased. In the present study, when the replacement level was increased from 0 to 20%, there were no significant differences in fat and protein digestibility among the experimental groups, whereas the replacement over 30% digestibility was significantly lower. This was in line with the pattern of effects on nutrient digestibility of most plant protein sources replacing FM (77–79). The excessive addition of CGM to the feed resulted in reduced digestibility, mainly due to ANF and AA imbalances (53). In addition, the replacement of small amounts (20%) of FM with CGM significantly improved digestibility of dry matter and energy in *L*. *vannamei*. This was in line with the study on *M. rosenbergii* (56), indicating that small amounts of CGM were well-digested and absorbed by aquatic animals.

Feed amino acid digestion and absorption were among the most important factor influencing the formulation of efficient shrimp feeds. The digestibility of AA as an indicator to evaluate the quality of feeds has received increasing attention (80). The digestibility of raw protein depends on its amino acid composition and absorption efficiency, and a deficiency in any of the essential amino acids will result in reduced protein utilization. In the present study, the digestibility of each amino acid was also largely above 78%, in line with the study by Akiyama et al. (81). A study by Fox et al. (82) showed that the main limiting amino acids in *L*. *vannamei* were Lys, Met, and Arg, considered to be Lys > Met > Arg in that order of importance. In the present study, the difference in digestibility of AA in *L*. *vannamei* was insignificant between 0, 10, and 20% of FM replacement with CGM, and the digestibility of the AA reached over 82.98%. Similar results were obtained in studies on *Sparus auratus* (83, 84), *Salmo salar* (85), and *Melanogrammus aeglefinus L*. (86). It indicated that CGM was an excellent protein source that could replace small amounts of FM, as it contained only small amounts of fiber and ANF so aquatic animals could be better digested and absorbed by them. However, in the present study, the digestibility of Lys and TAA in *L*. *vannamei* was significantly reduced when the proportion of CGM replacement exceeded 30%, in line with the protein digestibility. Masumoto et al. (87) reported that the apparent digestibility of TAA from CGM was poor in *Seriola quinqueradiata* and significantly lower than that of FM. NEAA are those that can be synthesized in the animal's body and meet its needs for maintenance, growth, development and health without the need to be supplied by the diet. In the present study, the digestibility of NEAA (except for serine) was significantly reduced for all replacement levels above 30%. The speculated reason is that the high proportion of CGM has caused an extreme imbalance of AA in the feed, leading to a reduction in AA digestibility. Therefore, it is not advisable to replace too high a proportion of FM with CGM. This could affect the digestion of AA, which may in turn affect protein synthesis and the growth of aquatic animals.

## 5. Conclusion

In conclusion, the partial replacement of FM (20%) with CGM in shrimp feed significantly improved the apparent digestibility of dry matter and energy. Based on the results obtained in growth performance and digestive enzyme activity, it is concluded that replacing FM with CGM in the diet of juvenile white shrimp, may be an advisable choice. Based on the broken-line regression analysis of WGR, the optimal dietary CGM replacement was 27.47%.

## Data availability statement

The original contributions presented in the study are included in the article/supplementary material, further inquiries can be directed to the corresponding authors.

## Ethics statement

This study followed the recommendations set-out by the Care and Use of Laboratory Animals in China from the Animal Ethical and Welfare Committee of China Experimental Animal Society. The protocol was approved by the Animal Ethical and Welfare Committee of Guangdong Ocean University (Guangdong, China), processing ID GDOU-AEWC-20180063.

## Author contributions

HL and YD was responsible for breeding experiments, data analysis, and article writing. DZ and LF was responsible for the purchase of experimental consumables. QY was responsible for directing experiments, paper revisions, and funding acquisition. XZ was responsible for the maintenance of laboratory equipment. BT was responsible for experimental data guidance and funding acquisition. SC was responsible for the guidance of breeding experiments. QY, XZ, and HL reviewed and revised the manuscript. All authors contributed to the article and approved the submitted version.
